# Outlier concepts auditing methodology for a large family of biomedical ontologies

**DOI:** 10.1186/s12911-020-01311-x

**Published:** 2020-12-15

**Authors:** Ling Zheng, Hua Min, Yan Chen, Vipina Keloth, James Geller, Yehoshua Perl, George Hripcsak

**Affiliations:** 1 Computer Science and Software Engineering Department, Monmouth University, West Long Branch, NJ 07764 USA; 2 Department of Health Administration and Policy, George Mason University, Fairfax, VA 22030 USA; 3 CIS Department, Borough of Manhattan Community College, CUNY, New York, NY 10007 USA; 4 Department of Computer Science, New Jersey Institute of Technology, Newark, NJ 07102 USA; 5 Department of Biomedical Informatics, Columbia University, New York, NY 10032 USA

**Keywords:** Biomedical ontologies, Ontology quality assurance, Auditing BioPortal ontologies, Ontology auditing scalability, Summarization network, Ontology error concentration, Meta-ontology

## Abstract

**Background:**

Summarization networks are compact summaries of ontologies. The “Big Picture” view offered by summarization networks enables to identify sets of concepts that are more likely to have errors than control concepts. For ontologies that have outgoing lateral relationships, we have developed the "partial-area taxonomy" summarization network. Prior research has identified one kind of outlier concepts, concepts of small partials-areas within partial-area taxonomies. Previously we have shown that the small partial-area technique works successfully for four ontologies (or their hierarchies).

**Methods:**

To improve the Quality Assurance (QA) scalability, a family-based QA framework, where one QA technique is potentially applicable to a whole family of ontologies with similar structural features, was developed. The 373 ontologies hosted at the NCBO BioPortal in 2015 were classified into a collection of families based on structural features. A meta-ontology represents this family collection, including one family of ontologies having outgoing lateral relationships. The process of updating the current meta-ontology is described. To conclude that one QA technique is applicable for at least half of the members for a family *F*, this technique should be demonstrated as successful for six out of six ontologies in *F*. We describe a hypothesis setting the condition required for a technique to be successful for a given ontology. The process of a study to demonstrate such success is described. This paper intends to prove the scalability of the small partial-area technique.

**Results:**

We first updated the meta-ontology classifying 566 BioPortal ontologies. There were 371 ontologies in the family with outgoing lateral relationships. We demonstrated the success of the small partial-area technique for two ontology hierarchies which belong to this family, SNOMED CT’s *Specimen* hierarchy and NCIt’s *Gene* hierarchy. Together with the four previous ontologies from the same family, we fulfilled the “six out of six” condition required to show the scalability for the whole family.

**Conclusions:**

We have shown that the small partial-area technique can be potentially successful for the family of ontologies with outgoing lateral relationships in BioPortal, thus improve the scalability of this QA technique.

## Background

Biomedical ontologies are essential for biomedical information systems and for their interoperability [1–5]. They are also critical for biomedical research, e.g., phenotyping with EHR text [3, 6–9]. The size of an ontology may be defined as the number of its concepts. The complexity of an ontology is measured by the ratio of the number of relationships connecting the concepts to the number of concepts. Most widely used ontologies are large and complex. This is apparent when looking at the most accessed ontologies in the BioPortal [10] of the National Center for Biomedical Ontologies (NCBO) [11] at Stanford University. For example, the National Cancer Institute Thesaurus (NCIt) [12], a cancer-focused ontology, has 138,291 concepts and 569,810 relationships in the March 2018 release, which results in an approximate complexity of 4.12. The most accessed ontologies include SNOMED CT [13], GO [14] and ChEBI [15].

Due to the size and complexity of ontologies, modeling errors and inconsistencies are unavoidable. It is important to correct errors in ontologies to prevent their propagation into the biomedical information systems using these ontologies. There is extensive research on quality assurance (QA) of ontologies [16–18], resulting in various automatic/semi-automatic methods to improve the quality of ontologies. Due to limited human resources, it is not practical to audit all the concepts of an ontology. Thus, one approach in QA of ontologies is to identify sets of concepts with a higher likelihood of errors than control samples. An example of such a methodology is based on identifying non-lattice structures in the hierarchy of an ontology [19–22]. Another framework, comprising several methodologies, was developed based on summarization networks. The Structural Analysis of Biomedical Ontologies Center (SABOC) [23] team has developed different summarization network-based QA techniques for many biomedical ontologies, e.g., for GO [24, 25], SNOMED CT [26–32], and NCIt [33–36] (Please refer to Table 1 for the terms used in the following writing).

**Table 1:** Glossary

Term	Definition	Example
*is-a* relationship	The subsumption relationship underlying the hierarchy of an ontology is called *is-a* relationship	A hierarchical *is-a* relationship connecting the concept *Regulatory Gene* to the concept *Gene* in Fig. 1a
Lateral relationship	The non-hierarchical semantic relationship is called lateral relationship, in contrast to the hierarchical *is-a* relationship. It is called “role” in NCIt and “attribute relationship” in SNOMED CT	The NCIt concept *Antigen Gene* in Fig. 1a is defined by its lateral relationship (or role) *Gene Plays Role In Process* with the value *Immune Response Process*
Area	An area is a group of all the concepts having exactly the same set of lateral relationship types	Figure 1b has an area colored in blue and labeled as *Gene Plays Role In Process*, summarizing four concepts
Partial-area	A partial-area is a subunit in an area defined by a root concept describing the semantic of the partial-area, including also its all descendant concepts within the area sharing the same semantic	Figure 1c has a partial-area labeled as *Antigen Gene (4)* in the right blue area
Small partial-area	A partial-area is small if its size is not larger than a bound b, where b is a small number, typically lower or equal to 10	The partial-area *MicroRNA Gene (2)* in the left blue area in Fig. 1c is a small partial-area with size 2

Summarization network-based QA techniques start with the derivation of summarization networks for ontologies. Such networks are composed of nodes and hierarchical links connecting them, in which a node represents a set of similar concepts. Hierarchical links are derived based on the hierarchical *is-a* relationships between concepts. Hence, summarization networks are compact summaries of ontologies. Summarization networks are derived by algorithms based on structural features of the ontologies.

Different ontologies may have different structural features, thus they will have different kinds of summarization networks and different definitions of similarity among concepts. For example, concepts in eight of the 19 hierarchies of SNOMED CT have outgoing lateral relationships, while concepts in the remaining 11 hierarchies only serve as targets of lateral relationships from eight other hierarchies. Two kinds of summarization networks have been developed for these two different kinds of hierarchies: partial-area taxonomies [30] and Tribal Abstraction Networks (TANs) [27] respectively. In a partial-area taxonomy, the nodes are partial-areas, which summarize sets of concepts with exactly the same set of lateral relationships that are all hierarchically under one specific root concept. The root concept provides the partial-area its name and semantics [37].

The “Big Picture” ontology view offered by summarization networks enables users to identify sets of concepts that are more likely to have errors than control concepts. Such sets can be utilized to guide curators of ontologies to concentrate on concepts for which a better QA yield can be achieved. The yield is measured by the ratio of the number of identified errors to the number of reviewed concepts. Two themes that have been shown to typically indicate higher concentrations of errors than found in control samples are *complex concepts* [33, 38] and *uncommonly modeled concepts* [26, 38].

Most research on QA techniques has been demonstrated to be effective for individual ontologies. To improve QA scalability, He et al. [39] and Ochs et al. [40] developed a family-based QA framework where one QA technique is potentially applicable to a whole family of ontologies with similar structural features. They classified the 373 ontologies hosted at that time at the NCBO BioPortal [10], the largest existing ontology repository, into a collection of families based on structural features. A meta-ontology [40] was used to represent this family collection. For example, there were 279 ontologies in the family where concepts have outgoing lateral relationships. Lateral relationships are an essential feature to derive partial-area taxonomies.

In order to conclude that one QA technique is potentially applicable for a family *F*, this technique should be demonstrated as successful on six out of six ontologies in family *F* [40]. Then this technique will be applicable to at least half of the ontologies in *F*. For example, if a family *F* has 20 ontologies and one technique is successful for six of its ontologies, then it is guaranteed to be applicable for at least 10 ontologies of *F*.

One of the techniques falling under the above theme of complex concepts is the set of overlapping concepts. Overlapping concepts are concepts which belong to multiple partial-areas in a partial-area taxonomy of an ontology. The exact specification of overlapping concepts is complex and required the definition of a refinement of the partial-area taxonomy summarization network into the disjoint partial-area taxonomy summarization network [41]. We have shown that the *overlapping complex concepts-based* technique is potentially applicable to a family of 76 ontologies with two features, (1) having outgoing lateral relationships and (2) [some] concepts having multiple parents [33].

In a long-range research program, the SABOC team has repeatedly demonstrated that one specific kind of uncommonly modeled concepts, namely *concepts in small partials-areas* within partial-area taxonomies, are statistically significantly more likely to have errors than sets of concepts in large partial-areas. The small partial-area technique was previously shown to work successfully for four ontologies (or hierarchies in ontologies). They are the NCIt’s *Neoplasm* subhierarchy [35], the *Biological Process* hierarchy [37], SNOMED CT’s *Procedure* hierarchy [29], and the Chemical Entities of Biological Interest (ChEBI) [42] ontology [43]. Note that since different hierarchies in SNOMED CT and NCIt were developed and maintained by different teams with different features, we cannot assume that if a technique works for one hierarchy in such an ontology, it will necessarily work for another hierarchy. Thus, we have considered each hierarchy in these two ontologies as an individual ontology.

Can this technique be potentially successful for the whole family of ontologies with outgoing lateral relationships? For an affirmative answer, we need to show its success on six out of six ontologies. Hence, in this paper, we investigate this technique on two more ontologies: SNOMED CT’s *Specimen* hierarchy and NCIt’s *Gene* hierarchy, which belong to the same family as the previous four ontologies.

In the time passed since the previous research [40], the number of ontologies in the NCBO BioPortal has increased as of August 2019 to 796. Thus, we will update the meta-ontology of the families of BioPortal ontologies [40] to the current situation. This will increase the impact of the applicability of the small partial-areas and the overlapping concepts techniques beyond the 279 ontologies (now 371) and 76 ontologies of the previous study [40], according to the newer collection of ontologies in BioPortal. Finally, we received queries from readers of previous papers [33] and [40] requesting the details of the statistical analysis leading to the result of six out of six. Thus, we include in this paper the detailed analysis which did not appear before.

The two ontologies analyzed in this paper are SNOMED CT [13] and the National Cancer Institute Thesaurus (NCIt) [12]. Before providing the background for each of them, we first describe their common properties. SNOMED CT and NCIt are arguably the two most important and frequently used clinical ontologies in biomedicine. Both are modeled by a version of description logic, thus the basic building blocks are concepts that are connected by *is-a* relationships forming a hierarchy.

In a hierarchy, a concept may have multiple parent concepts, i.e., multiple *is-a* relationships pointing upward. (We are using the simpler term "hierarchy," as opposed to other terms in use in the community, such as "heterarchy.") Hence, the hierarchy can be presented as a directed acyclic graph (DAG). In contrast to the *is-a* hierarchical relationship, a lateral semantic relationship connects two concepts, which may be in different hierarchies to specify a defining characteristic of the source concept. Each lateral relationship has a specified domain (i.e., the source hierarchy in which a lateral relationship can be applied) and a corresponding range (i.e., the target hierarchy to which the lateral relationship can point). Note that not every hierarchy serves as domain (i.e., not every hierarchy has been defined with lateral relationships); instead, some hierarchies serve only as ranges of lateral relationships.

Lateral relationships are inherited from parent concepts to child concepts. For example, the concept *Neoplasm of digestive system* in SNOMED CT has an *is-a* relationship to the concept *Disorder of digestive system* and a lateral relationship named *Finding site* pointing to the target concept *Structure of digestive system*. The lateral relationship is inherited by the concept *Malignant neoplasm of digestive system* which is a child concept of *Neoplasm of digestive system*. Both SNOMED CT and the NCIt have an asserted and an inferred release. The asserted release contains assertions explicitly defined by the curator team, while the inferred release is obtained by running a reasoner on the former one. In this paper, we used the inferred releases of SNOMED CT and NCIt.

### SNOMED CT

SNOMED CT [44] is the most comprehensive, multilingual clinical healthcare ontology in the world, which is in use in more than eighty countries and is now accepted as a common global standard for health terms. It includes terms for a wide range of clinical specialties, disciplines and requirements. Thus, it enables the accurate recording and sharing of clinical and health information and facilitates the semantic interoperability of Electronic Health Records [45]. It is maintained and distributed by SNOMED International [46]. There are two new releases of the SNOMED CT International Edition in each year, released in January and in July, respectively. SNOMED CT is released in tab-delimited flat files. In this paper, we utilized the January 2018 release of the SNOMED CT International Edition.

SNOMED CT’s concepts are divided into 19 hierarchies (e.g., *Clinical Finding* and *Specimen*). Lateral relationships are called attribute relationships in SNOMED CT. Among the 19 hierarchies, eight hierarchies are defined with attribute relationships and the other 11 hierarchies serve only as ranges of attribute relationships, e.g., *Organism*. In the January 2018 release, there were 341,105 concepts connected by 511,767 *is-a* hierarchical relationships and 550,307 attribute relationships. For the *Specimen* hierarchy considered in this study, there were 1696 concepts defined by five types of attribute relationships, i.e., *Specimen source topography* (1334 concepts), *Specimen procedure* (902 concepts), *Specimen substance* (774 concepts), *Specimen source morphology* (147 concepts), and *Specimen source identity* (118 concepts).

### National Cancer Institute Thesaurus (NCIt)

The National Cancer Institute Thesaurus (NCIt) [12] is an ontology focused on cancer related information, including clinical care, translational and basic research, and public and administrative information. It is widely used by various information systems at the National Cancer Institute (NCI) and outside of NCI, nationally and internationally. NCIt facilitates interoperability and data sharing in the cancer research community [47]. NCI manages and publishes the NCIt monthly through NCI Enterprise Vocabulary Services (EVS) in OWL and flat file formats. The NCIt can be accessed through the NCI Term browser [48]. Lateral relationships are called roles in NCIt. We will use "relationships" from this point on to refer to lateral relationships for both ontologies.

The NCIt’s March 2018 release used in this paper had 138,291 concepts organized into 19^1^ disjoint IS-A hierarchies and connected by 148,460 *is-a* hierarchical relationships and 421,350 roles. Examples of the hierarchies are *Disease Disorder or Finding*; *Gene*; *Biological Process*; *Molecular Abnormality*; and *Abnormal Cell*. There are 11 hierarchies defined with relationships, e.g., *Gene* and *Biological Process*, and eight hierarchies serving only as targets of relationships, e.g., *Organism* and *Biochemical Pathway*. The *Gene* hierarchy investigated in this research had 10,117 concepts at the time, which was almost six times the number of concepts in the *Specimen* hierarchy of SNOMED CT.

The *Gene* hierarchy is defined with 16 types of relationships, including the following five most frequent relationships *Gene Plays Role In Process* (9325 concepts), *Gene In Chromosomal Location* (3722 concepts), *Gene Found In Organism* (3359 concepts), *Gene Is Element In Pathway* (2457 concepts), and *Gene Associated With Disease* (1365 concepts).

### Partial-area taxonomy

In a long-range research program by the Structural Analysis of Biomedical Ontologies Center (SABOC), summarization networks have been developed and applied to QA of ontologies. They enable to characterize subsets of concepts that are statistically significantly more likely to have errors [38] than concepts in a random control group. A summarization network is a network of *nodes* connected by hierarchical *child-of* links. Each node summarizes a group of similar concepts. Compared to an ontology itself, the summarization network, derived from it, is more compact. Two typical summarization networks are called *area taxonomy* and the *partial-area taxonomy* [30].

The nodes in an *area taxonomy*, automatically derived from an ontology, are called *areas*. An area is a group of all the concepts having exactly the same set of relationship types. Each concept can be summarized by exactly one area, according to its type(s) of relationships. Hence, areas are disjoint. Areas are labeled by their set of relationship types with the number of concepts that they summarize. A *root* concept of an area is a concept such that all its parent concept(s) are not in this same area. An area may have multiple root concepts. *Child-of* links connecting areas are derived from the hierarchical *is-a* relationships between concepts in the ontology. Namely, if a root concept of an area **A** has a parent concept in another area **B**, then area **A** is *child-of* area **B.**

**Fig. 1:**
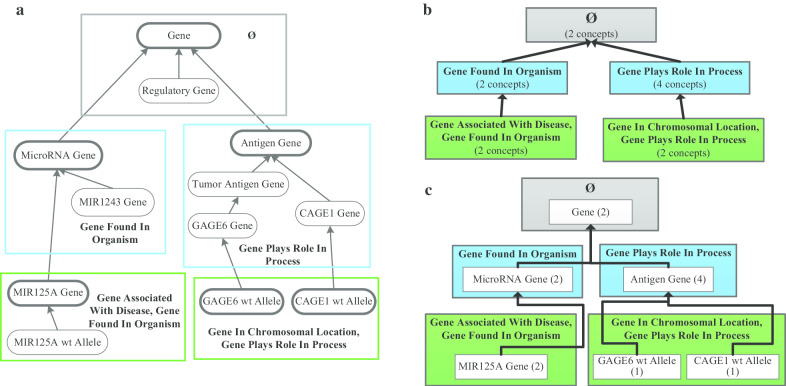
**a** An excerpt of 12 concepts from NCIt’s *Gene* hierarchy. Concepts are denoted by round-corner boxes and are connected by *is-a* relationships represented by upward arrows. Colored rectangles enclose concepts with the same set of relationship types (in bold). Root concepts are shown as bold boxes. **b** The area taxonomy for **a**. Areas are presented as colored boxes based on the number of relationship types, i.e., areas with the same number of relationship types have the same color. An area is labeled by the set of its relationship types and the number of concepts that it summarizes in parentheses. Areas are connected by *child-of* links shown as bold upward arrows. **c** The partial-area taxonomy for **a**. Partial-areas are shown as white boxes inside areas. A partial-area is labeled by its root concept and the number of concepts that it summarizes in parentheses. Partial-areas are connected by *child-of* links represented as bold arrows, as in the area taxonomy

Figure 1b shows the *area taxonomy* derived for an excerpt of 12 concepts from NCIt’s *Gene* hierarchy in Fig. 1a. For example, in Fig. 1a, the two concepts *MicroRNA Gene* and its child concept *MIR1243 Gene* enclosed in the left blue rectangle have only one relationship type *Gene Found In Organism.* Hence, they are represented as the left blue area in Fig. 1b, labeled as *Gene Found In Organism* (2 concepts). The concept *MicroRNA Gene* is the root concept of the area, because its parent concept *Gene* is in another area. The latter area has no relationships and hence is labeled as Ø (= the empty set). As a result, the area *Gene Found In Organism* (2 concepts) has a *child-of* link (indicated by the bold upward arrow) pointing to the area Ø, which is called the root area of this area taxonomy.

If an area has multiple root concepts, then it includes concepts with different semantics, represented by the different root concepts. For example, in Fig. 1a there are two root concepts *GAGE6 wt Allele* and *CAGE1 wt Allele* in the right green area, representing two different genes*.*

To obtain groups of concepts having both similar structure and similar semantics, an area is divided into *partial-area*(s). A *partial-area* consists of a root concept and all its descendant concepts in the same area, which are sharing the same semantics represented by the root concept. Thus, a partial-area is labeled by its root concept and the number of concepts in the partial-area. Partial-areas are connected by *child-of* links to form a partial-area taxonomy. Similar as in the area taxonomy, if the root concept of partial-area **A** has a parent concept in partial-area **B**, then **A** is *child-of* **B**. Figure 1c shows the partial-area taxonomy for Fig. 1a. For example, the right green area is divided into two partial-areas and the partial-area *GAGE6 wt Allele (1)* is *child-of* the partial-area *Antigen Gene (4)*. *Gene* is the only root concept of the area Ø and the partial-area *Gene (2)* is the root of the partial-area taxonomy.

### Related partial-area taxonomy-based quality assurance studies

The SABOC team has conducted and published many QA studies [49] successfully utilizing summarization networks of ontologies to identify characterizations of concepts more likely to have errors. Two repeated themes among these studies are (1) *complex concepts* and (2) *uncommonly modeled concepts*. Examples of complex concepts are *overlapping concepts* [32, 33] and *concepts with many relationship types* [50, 51]. *Concepts in small partial-areas* of partial-area taxonomies [37] and *concepts forming a large area without any relationships* [34] are two examples of uncommonly modeled concepts. Some of these concepts, e.g., overlapping concepts and concepts in small partial-areas, can only be seen through "the lens" of a partial-area taxonomy. The previous four successful QA studies on concepts in small partial-areas are described as follows.

In the study on NCIt’s *Neoplasm* subhierarchy [35], we found that the error rates of concepts in small partial-areas (size ≤ 10) are twice as big as error rates for large partial-areas. This was shown with statistical significance (the *p* value of Fisher’s exact test is less than 0.05). Hua et al. [37] reported a study on NCIt’s *Biological Process* hierarchy, in which the percentage of erroneous concepts in partial-areas with three or fewer concepts (12%) is higher than for other concepts (5%). Although they did not report the *p* value, based on their reported data, we calculated the *p* value of Fisher’s exact test as 0.0011 (< 0.05), meaning concepts in small partial-areas (size ≤ 3) have statistically significantly more errors than concepts in partial-areas with sizes greater than three.

In the study on SNOMED CT’s *Procedure* hierarchy by Ochs et al. [29], the small partial-areas (size ≤ 3) were reported to harbor more errors than large partial-areas, with statistical significance (*p* = 0.019 < 0.05). Liu et al. [43] investigated the small partial-area error concentration of the chemical ontology ChEBI and obtained statistical significance (*p* = 0.0003) for the comparison of error rates between small (size ≤ 2) and large partial-areas. For all four cases, concepts of small partial-areas have statistically significantly more errors than concepts of large partial-areas, although the interpretation of “small” varies.

### BioPortal ontologies

BioPortal, a website maintained by the National Center for Biomedical Ontology located at Stanford, is widely considered to be the world's most comprehensive repository of biomedical ontologies (https://bioportal.bioontology.org/). Since its inception it has been growing on a regular basis, reaching 860 ontologies with over 11 million classes (~ concepts) as of May 2020. In addition, BioPortal provides tools such as an annotator program (in beta release) and an ontology recommender and usage statistics for individual ontologies. The latter include monthly visits and individual projects using a specific ontology. BioPortal is regularly updated with the most recent release of an ontology, with earlier releases being archived. As to the exact definition of what qualifies as a biomedical ontology, BioPortal is agnostic. Terminologies that are of relevance to biomedicine are included, even if they do not pass muster according to diverse definitions of what it means to be an ontology.

## Methods

### Updating the meta-ontology for BioPortal ontologies

Ochs et al. [40] introduced a meta-ontology describing various structure-based families of ontologies appearing in BioPortal. These families covered 373 out of 439 ontologies hosted in BioPortal at a point in 2015. Meanwhile the collection of ontologies in the BioPortal grew to 796 (as of 8/29/2019). We are presenting in this paper a meta-ontology updated to reflect the current situation. This update will enable us to report the current number of ontologies in the family of *ontologies with relationships* for which the QA methodology of small partial-areas is applicable. Similarly, we will be able to update the number of ontologies in the family of *DAG ontologies with relationships* for which the overlapping concepts QA methodology [33] is applicable.

**Fig. 2:**
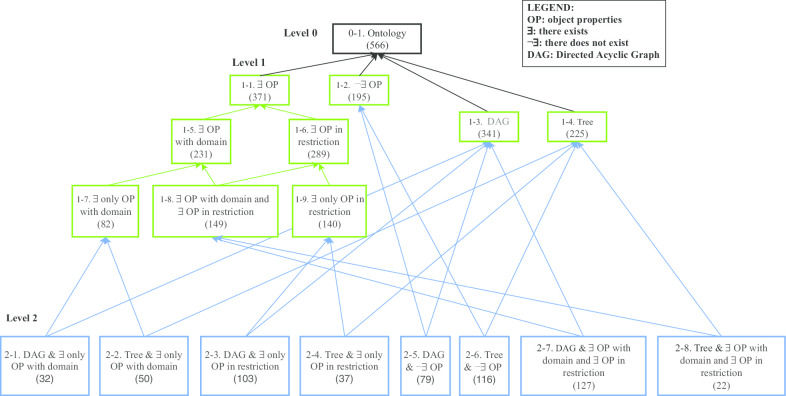
The structured-based meta-ontology for BioPortal ontologies in August 2019

The BioPortal-based meta-ontology [40] categorizes the stored ontologies into families based on the structural features of the ontologies, namely (1) object-properties (OP) (~ relationships), (2) data-properties (DP) (~ attributes), and (3) hierarchy structure (Is it a tree or a DAG?). Since our current QA methodologies do not involve data-properties, we will present the meta-ontology without the DP category, thus simplifying the diagram (Fig. 2). This diagram will incorporate the numbers of ontologies for which the *small partial-area* QA methodology and the *overlapping concepts* QA methodology are applicable.

According to previous work [40], out of 373 ontologies in BioPortal in 2015, there were 279 ontologies having the structural feature "outgoing lateral relationships," including the six ontologies analyzed in this paper. Establishing the success of the QA methodology based on small partial-areas (which relies on outgoing lateral relationships) for two more ontology hierarchies in this paper implies that the small partial-area-based QA technique can be applied to this whole ontology family. QA for large, existing ontologies is considered beyond the available resources of most organizations. Thus, curators of the ontologies in this family could concentrate their available, limited auditing resources on concepts in small partial-areas within partial-area taxonomies, so that they would get a better QA yield than auditing a random sample of concepts of the same size.

### Is the small partial-area-based QA methodology applicable for a family of BioPortal ontologies?

To claim that a QA technique is potentially applicable to a whole family of ontologies, this technique should be demonstrated being successful on six out of six ontologies or on eight out of nine ontologies. The rationale of this statement is as follows.

We consider whether a QA technique is working for an ontology or not as an independent experiment. The experiments on a list of ontologies from the same family have a series of binary outcome, i.e., working (success) with a probability *p* or not working (failure) with a probability (1 - *p*), following a binomial distribution.

$$\left(\begin{array}{c}n\\ i\end{array}\right){p}^{i}{(1-p)}^{n-i}$$

The reason is that for a specific sequence with *i* successes and (*n* - *i*) failures the probability is ${p}^{i}{(1-p)}^{n-i}$. There are $$\left(\begin{array}{c}n\\ i\end{array}\right)= \frac{n!}{i!\left(n-i\right)!}$$ ways to select a specific sequence with *i* successes and (*n* - *i*) failures yielding $$(\begin{array}{c}n\\ i\end{array}){p}^{i}{(1-p)}^{n-i}$$. We need to test whether the observed experimental results are likely to have been generated by chance alone, assuming equal probability for each state and using 0.05 as our threshold for statistical significance.

Given the small numbers of ontologies in each family, we calculate exact confidence intervals (as opposed to normal approximations) and—to be conservative—we use central confidence intervals (as opposed to Stern’s narrower but asymmetric confidence intervals). Specifically, we use the exact binomial central confidence intervals defined by Clopper and Pearson [52]. In experiments where all the items are in the same state (i.e., success), six is the minimum number to achieve statistical significance. That is, with six out of six successes, the 95% confidence interval on the underlying probability is 0.541–1, which excludes chance or 0.5. That means, if a QA technique is demonstrated successful on six out of six ontologies, it will also be successful for other ontology members in the same family with a probability between 0.541 and 1. That is, for at least half of the ontologies in this family, this QA technique is likely to be successful.

In experiments where one differs, nine is the minimum number to achieve statistical significance. That is, with eight out of nine successes, the 95% confidence interval on the underlying probability is 0.518 to 0.997, again excluding 0.5. Twelve (10 out of 12) achieves significance if two differ from the others, and so on. In all these cases, the technique is likely to be successful for at least half of the ontologies in this family.

As described in Background, we already have four successful studies showing that concepts in small partial-areas are statistically significantly more likely to have errors than concepts in large partial-areas. The definition of “small” varies for different ontologies in this paper. In order to achieve six successes, we conducted QA studies on SNOMED CT’s *Specimen* hierarchy and NCIt’s *Gene* hierarchy, since they belong to the same structural family as the previous four successful ontologies. The following hypothesis was investigated in the two QA studies.

#### **Hypothesis 1**

Concepts in small partial-areas of the partial-area taxonomy derived from an ontology have statistically significantly more errors than concepts in large partial-areas.

Concepts in a partial-area share similar structure and semantics. The reason why small partial-areas harbor more errors is that the concepts in small partial-areas probably appear there due to uncommon modeling. These concepts are considered as outlier concepts, since in the whole ontology there are only a few concepts with the combination of the specific structure and semantics as of this small partial-area. This uncommon modeling may have resulted from modeling errors in the ontology. Once these errors are corrected, concepts in small partial-areas will likely be merged into big(ger) partial-areas.

Consider, for example, the concept *Tendon biopsy sample*. In the January 2018 SNOMED CT release, it has two parent concepts, *Tendon sample* and *Biopsy sample*, thus *Tendon biopsy sample* itself is a partial-area of one single concept. However, in the January 2019 release, its parent concept *Biopsy sample* was replaced by the concept *Soft tissue biopsy sample*, resulting in *Tendon biopsy sample* being moved into the partial-area *Soft tissue biopsy sample* containing 22 concepts. We consider this change as a correction of a modeling error that existed in the January 2018 release. Through this example it becomes clear that corrections of modeling errors can simplify the structure of ontologies, which is reflected in a reduced number of concepts in small partial-areas, i.e., outlier concepts. Similar simplifications were shown in previous work [33, 53].

**Fig. 3:**
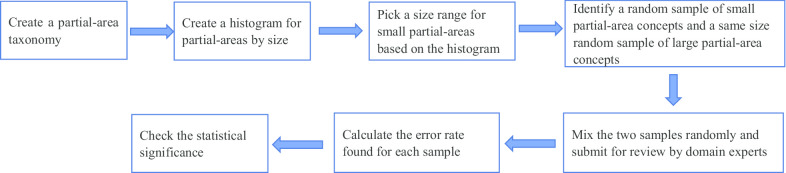
The flow chart summarizing the process of the small partial-area based QA study

The following flow chart (Fig. 3) summarizes the process of the study to show success of applying the small partial-area based QA methodology on an ontology.

### QA methodology for the SNOMED CT *Specimen* hierarchy

To investigate Hypothesis 1 on the *Specimen* hierarchy of SNOMED CT, we conducted a randomized control trial on a sample of specimen concepts in SNOMED CT. In order to obtain the sample, the partial-area taxonomy was first automatically derived from the *Specimen* hierarchy of the SNOMED CT January 2018 release using the software tool Ontology Abstraction Framework (OAF) [54] developed by the SABOC team. One kind of concepts named “overlapping concepts” in partial-area taxonomies have been demonstrated prone to have more errors than non-overlapping concepts [33]. To avoid biasing the results, overlapping concepts were excluded from this study. According to our previous experience, the exact threshold to distinguish between “small” and “large” for different ontologies varies and is determined by the study's results. Thus, we initially consider partial-areas with the number of concepts (i.e., size) ranging from 1 to 10 as small partial-areas and partial-areas with more than 10 concepts as large partial-areas.

Utilizing the derived partial-area taxonomy, we collected a random sample of 100 specimen concepts, consisting of 50 concepts from small partial-areas and 50 concepts from large partial-areas. To investigate the preferred threshold of “small” partial-areas for the *Specimen* hierarchy, for each size ranging from 1 to 10, the number of chosen concepts was proportional to the total number of concepts with this size. The small partial-area concepts and large partial-area concepts were mixed into a list with a random order. The domain expert, YC, who has medical and ontological training and extensive QA experience on biomedical ontologies, reviewed this list of 100 random concepts to check whether there are modeling issues for each one and recorded the suggested corrections.

The study hypothesis was unknown to YC. YC also had no idea which concept is from a small partial-area and which concept is from a large partial-area. Based on her error report, we first determined the best threshold of partial-area size to distinguish between small partial-areas and large partial-areas for the *Specimen* hierarchy. Then we calculated the two-tailed *p* value of Fisher’s exact test [55] to investigate whether there is a statistically significant difference between the error rates of small partial-area concepts and large partial-area concepts.

### QA methodology for the NCIt *Gene* hierarchy

The QA methodology for the NCIt *Gene* hierarchy is similar as that for the SNOMED CT *Specimen* hierarchy. First, we derived the partial-area taxonomy for the *Gene* hierarchy of the NCIt March 2018 release using the OAF software tool. Then we randomly chose 50 concepts from small partial-areas and 50 concepts from large partial-areas. At this step, overlapping concepts in the partial-area taxonomy were excluded to avoid bias.

The difference between the two studies is the sampling technique from the small partial-area concepts due to the large difference between the numbers of concepts in the two hierarchies. As mentioned in the Background section, the *Gene* hierarchy of NCIt is six times larger than the *Specimen* hierarchy of SNOMED CT. For the *Gene* hierarchy, for each size of small partial-area, ranging from 2 to 10, five concepts were randomly picked. Since the number of partial-areas with size = 1 is much larger than that of other small partial-areas, 10 concepts were randomly chosen. The reason for this approach to sampling is the need to represent different sizes fairly.

The randomly mixed 100 concepts were presented to the domain expert, HM, who is trained in medicine and biomedical ontologies and has conducted extensive QA studies on NCIt. Similar to the study on the *Specimen* hierarchy, HM was blinded to the study hypothesis to avoid bias. Furthermore, she did not know which concepts are from small partial-areas. After reviewing the sample, HM submitted an error report on observed modeling issues with suggested corrections. Again, the preferred threshold for the size of small partial-areas was selected based on the error percentages. Then the two-tailed *p* value of Fisher’s exact test was calculated to evaluate the statistical significance of the hypothesis.

## Results

### Updated meta-ontology for BioPortal ontologies in August 2019

The theory underlying the structure-based meta-ontology is complex and was described at great length before [40]. The meta-ontology presented in Fig. 2 should be self-explanatory with the help of the legend. In this meta-ontology only 566 ontologies out of 796 ontologies in the BioPortal collection in August 2019 are presented. The remaining ontologies did not qualify for inclusion in the meta-ontology due to the following reasons. There are 74 ontologies without a submission file and 12 ontologies had license restrictions. No active URL existed for 21 ontologies and the rest of the ontologies that are not represented in the meta-ontology could not be parsed by the OWL API.

In the meta-ontology, in node 1–1., 371 ontologies have OPs (~ relationships), and therefore small partial-areas can be determined (if they exist). We note that the number of ontologies specified in the two children of 1–1. (1–5. and 1–6.) add up to more than 371. The reason is that those two families are not disjoint. A partition into disjoint families is achieved at the grandchildren of 1–1. (1–7., 1–8., and 1–9.).

### Results of the QA study on the SNOMED CT *Specimen* hierarchy

The partial-area taxonomy derived from the *Specimen* hierarchy with 1696 concepts in the SNOMED CT January 2018 release has 23 areas and 530 partial-areas. The sample of 100 concepts in the study was randomly selected from 1463 concepts, excluding overlapping concepts, as noted before.

Among the 100 reviewed concepts, the domain expert YC found 14 concepts (14%) having modeling issues. Table 2 shows the partial-area distribution and the concept distribution of the complete hierarchy, and the numbers of sample concepts and erroneous concepts for different partial-area sizes. For example, there are 345 partial-areas with only one concept. Among them, we randomly selected 22 concepts for review. The domain expert found that three of them (13.6% = 3/22) had modeling issues. Although there is a large error rate difference between the partial-areas with sizes smaller than 10 and those with sizes larger than or equal to 10, there is no trend of the error rates among partial-areas with sizes smaller than 10 discernible. Table 3 shows four example errors identified by the domain reviewer.

**Table 2:** The distribution of complete SNOMED CT specimen concepts, sample concepts and erroneous concepts by partial-area size

Partial-area size	# of partial-areas	# of concepts	# of sample concepts	# of erroneous concepts	Error percentage (%)
1	345	345	22	3	13.6
2	72	120	8	1	12.5
3	25	61	4	2	50.0
4	12	40	3	1	33.3
5	11	39	2	1	50.0
6	10	51	3	0	0
7	7	36	2	1	50.0
8	4	28	2	0	0
9	6	52	3	2	66.7
10	2	10	1	0	0
> 10	36	681	50	3	6.0
Total	530	1463	100	14	14

**Table 3:** Four examples of errors for SNOMED CT specimen concepts identified in the review

Concept	Partial-area size	Error	Suggested correction
Urethra biopsy sample	1	The target *body tissue material* of the attribute *Specimen substance* should be specific	Replace with *Urinary tract material*
Bursa tissue sample	2	Incorrect parent concept *Synovial tissue sample*	Change to *Tissue specimen*
Tissue specimen from eye	9	The target *body tissue material* of the attribute *Specimen substance* should be specific	Replace with *Eye tissue material*
Extradural lesion sample	22	The target *Morphologically abnormal structure* of the attribute *Specimen source morphology* should be specific	Replace with *lesion*

According to the erroneous concept percentage distribution in Table 2, we selected the partial-area size *nine* as the threshold to distinguish small partial-areas from large partial-areas, to achieve the maximum statistical significance of error rates (22.4% vs. 5.9%). The contingency table for the *p* value calculation between erroneous concepts from small partial-areas and from large partial-areas is shown in Table 4. The two-tailed *p* value of Fisher’s exact test is 0.0226, meaning that the difference of error rates between small partial-areas (size ≤ 9) and large partial-areas has statistical significance. In addition, the threshold 10 also has statistical significance with *p* value 0.0407. Hence, Hypothesis 1 was confirmed for the SNOMED CT *Specimen* hierarchy, resulting in the fifth successful study in the family of ontologies with outgoing lateral relationships.

**Table 4:** The 2 × 2 contingency table for erroneous small partial-area concepts and erroneous large partial-area concepts in the SNOMED CT *Specimen* hierarchy (with a two-tailed *p *value = 0.0226 < 0.05 by Fisher’s exact test)

	# Erroneous concepts	# Concepts w/o errors	Error percentage (%)
Small partial-areas (1–9)	11	38	22.4
Large partial-areas (≥ 9)	3	48	5.9

### Results of the QA study on the NCIt *Gene* hierarchy

The partial-area taxonomy derived from the *Gene* hierarchy with 10,117 concepts in the NCIt March 2018 release has 5594 partial-areas within 143 areas. The random sample of 100 gene concepts in this study was selected from 10,005 concepts excluding overlapping concepts in the partial-area taxonomy.

During the review on the 100 gene concepts, the domain expert HM found 62 concepts (62%) having modeling issues. Table 5 presents the results including the partial-area distribution and the concept distribution of the complete hierarchy, and the sample concept and erroneous concept distributions based on partial-area sizes. For example, in the partial-area taxonomy for the *Gene* hierarchy, there are 90 partial-areas with size = 2, that is, a total of 180 concepts. Five concepts out of them were randomly selected for review. The domain expert found four concepts (80% = 4/5) had modeling issues. As the partial-area size increases beyond two, there is no significant trend of error rates. However, the error rate for partial-area sizes one and two, is higher than that for the other sizes. Table 6 shows five example errors identified by the reviewer.

**Table 5:** The distribution of complete NCIt *Gene* concepts, sample concepts and erroneous concepts by partial-area size

Partial-area size	# of partial-areas	# of concepts	# of sample concepts	# of erroneous concepts	Error percentage (%)
1	5450	5450	10	9	90
2	90	180	5	4	80
3	4	12	5	1	20
4	5	20	5	3	60
5	2	10	5	3	60
6	1	6	5	3	60
7	2	14	5	2	40
8	2	16	5	1	20
10	1	9	5	3	60
> 10	37	4288	50	33	66
Total	5594	10,005	100	62	62

**Table 6:** Five examples of errors for NCIt *Gene* concepts identified in the review

Concept	Partial-area size	Error	Suggested correction
RBM5 wt Allele	1	Missing the relationship *Gene Associated With Disease* with the target *Lung Carcinoma*	Add the relationship
NUP98 Gene	1	Missing the relationship *Gene Plays Role In Process* with the target *DNA Replication*	Add the relationship
ZNF365 Gene	2	Missing the relationship *Gene Plays Role In Process* with the target *telomere maintenance*	Add the relationship
BCAR4 wt Allele	5	Missing the relationship *Gene Associated With Disease* with the targets *Breast Carcinoma* and *Cervical Carcinoma*	Add the two relationships
BRS3 Gene	654	Missing the relationship *Gene Associated With Disease* with the target *Lung Carcinoma*	Add the relationship

As before, we evaluated the statistical significance of error rate differences between small and large partial-areas by calculating the two-tailed *p* value of Fisher’s exact test using different thresholds. The results show that the partial-area size *two* is the threshold to distinguish between small and large partial-areas with the maximum statistical significance of error rates (86.7% vs. 57.6%). Table 7 illustrates the contingency table for the *p* value calculation using the threshold two, obtaining the two-tailed *p* value 0.043. That means that the error rate difference between small partial-areas (size ≤ 2) and large partial-areas has statistical significance. Thus, Hypothesis 1 was again confirmed for NCIt’s *Gene* hierarchy, resulting in the sixth successful study in the family.

**Table 7:** The 2 × 2 contingency table for erroneous small partial-area concepts and erroneous large partial-area concepts in the NCIt’s *Gene* hierarchy (with a two-tailed *p* value = 0.043 < 0.05 by Fisher’s exact test)

	# Erroneous concepts	# Concepts w/o errors	Error percentage (%)
Small partial-areas (1–2)	13	2	86.7
Large partial-areas (≥ 3)	49	36	57.6

## Discussion

Quality assurance of ontologies is an essential part of their life cycle [37]. Various techniques have been introduced to help with the auditing of ontologies. QA techniques are usually developed for individual ontologies. However, according to the family-based QA framework, it is possible that one technique is potentially applicable for a whole family of ontologies with similar structures. The condition for this is that such a technique is applied successfully to six out of six ontology members or eight out of nine ontology members of the same family. We had previously demonstrated that the technique of overlapping concepts in partial-area taxonomies, automatically derived from ontologies, can be applied to a whole family of 76 BioPortal ontologies [33].

In four ontologies (or hierarchies in ontologies), the concepts in small partial-areas of partial-area taxonomies have been shown more likely to have errors than concepts in large partial-areas. In order to demonstrate that this technique could be applied to the family of ontologies with outgoing lateral relationships, we presented studies on two more hierarchies of ontologies, SNOMED CT’s *Specimen* hierarchy and NCIt’s *Gene* hierarchy in this paper. The results of the two studies confirmed again the success of the small partial-area technique. Thus, this technique has achieved success for six out of six ontologies in this family with 371 ontologies. That means, the small partial-area technique can be applied successfully to at least half of the ontologies in this family, providing curators a QA methodology for these ontologies by focusing the limited QA resources on the small partial-area concepts in partial-area taxonomies.

Reviewing the six studies on small partial-area concepts, it becomes evident that the threshold of “small” partial-areas is different for various ontologies. This is not surprising, because both the size and the number of defined relationship types for each ontology differ. For example, the NCIt’s *Gene* hierarchy has five times more concepts than the SNOMED CT’s *Specimen* hierarchy. For the latter hierarchy, there are only five types of relationships while the former hierarchy has 16 relationship types. Furthermore, in the *Gene* hierarchy, there are many leaf concepts that represent a specific gene or its alleles. Since new relationships are defined for these leaves, each is represented in the partial-area taxonomy, as a partial-area of one concept. According to our long-term research, we did not encounter a threshold higher than 10 for the distinction between small and large partial-areas. Thus we defined a broad range from 1 to 10 as the boundary size to experiment with and find which value best distinguishes between small and large partial-areas.

Hence, while the phenomena of higher error rates for small partial-areas is broadly discernible, the border between small and large is flexible and needs to be determined by experimentation. For use with new ontologies in this family, curators are advised to mimic our research by experimenting first with a small sample of concepts from partial-areas of sizes 1–10. Based on the results, they can choose the threshold for this specific ontology and then audit the small partial-areas accordingly. Of course, if more QA resources are available, they should continue to audit (selected) large partial-areas also.

Furthermore, sometimes even within the sizes of the small partial-areas, there is a meaningful difference in the error rates. For example, for the *Specimen* hierarchy, the error rates for partial-area sizes 1 and 2 are about 13%, while for partial-area sizes 3–9, they are much higher. In this case, the curators are advised to start auditing with concepts in the partial-areas with sizes 3–9 before continuing with the concepts in partial-areas with sizes 1 and 2, as much as the available resources allow. Such an approach is expected to optimize the number of errors found for a given number of review hours.

Another way of prioritizing the review of the concepts in small partial-areas is by giving priority to those that also should have priority according to another technique such as "overlapping concepts" [33] or "high number of lateral relationships" [50]. For example, curators should first audit the overlapping concepts that belong to small partial-areas before advancing to the remaining small partial-area concepts.

In the *Gene* hierarchy more than half of the concepts are in partial-areas of one concept. Typically, there are no resources to audit about 5000 concepts. The challenge is to identify the most promising subset of these concepts for auditing with the available resources. From the derivation of the partial-area taxonomy in the Background section, we can see that when there are more defined relationship types for a hierarchy (or ontology), there are more possible combinations of relationship types. That means that there are more areas and more partial-areas in the derived partial-area taxonomy. Thus, the ratio of small partial-area concepts to all concepts is high. Dealing with this problem is left to future work and a potential solution is described below.

### Limitation

In this work we show that Hypothesis 1 is true for six out of six ontologies of the family of ontologies with lateral relationships. However, there is a problem in claiming that this condition implies that the hypothesis is true for at least half of the ontologies in the family. The problem is that the “success” of the hypothesis is defined as “have statistically significantly more errors.” The problem is with the need for statistical significance. To show statistical significance, the samples are required to have some minimum size.

If the number of lateral relationships in an ontology is very small, say two or three relationships, then typically the number of small partial-area concepts will be too small for a sample to show statistical significance. Also, if the total number of concepts in an ontology is not above some threshold, then again it would not be possible to show statistical significance even if the number of relationships is not small. Because the two samples, the study sample and the control sample, are not large enough to show statistical significance.

Thus, the conclusion of using the fact we show the truth of Hypothesis 1 for six out of six ontologies of this large family is only valid for the ontologies whose size and number of lateral relationships is large enough to enable to demonstrate statistical significance. Namely, at least half of ontologies having such conditions are guaranteed to satisfy Hypothesis 1.

### Future research

In previous work we have utilized the subtaxonomy constructed with a subset of relationship types to discover more overlapping concepts when the original partial-area taxonomy does not have enough overlapping concepts [36]. Here, we can utilize this kind of subtaxonomy technique to obtain fewer small partial-area concepts, i.e., to lower the ratio of small partial-area concepts to all concepts.

For example, if we use only the most frequent relationship type in the NCIt’s *Gene* hierarchy *Gene Plays Role In Process* (92.2% of gene concepts are defined with this relationship) to derive a subtaxonomy, there are only two areas and 686 partial-areas in the subtaxonomy. As a result, there are only 1105 concepts (10.9%) in partial-areas with sizes one and two, in contrast with the partial-area taxonomy with all relationship types where the number is considerably larger. For example, one of the concepts named *ENV* has been defined with two relationship types *Gene Found In Organism* and *Gene Plays Role In Process*. In the partial-area taxonomy considering all relationships this concept is in a partial-area containing only itself. However, if we use only the *Gene Plays Role In Process* relationship to create a subtaxonomy, it will be in the partial-area rooted at *Viral Gene* with 28 other concepts. Hence, the subtaxonomy is a promising technique to limit the number of small partial-areas if their number is quite large. In the future, we will conduct further research to experiment with such subtaxonomy technique for large hierarchies with many small partial-area concepts.

Another future direction will be to investigate the possibility of demonstrating success for six out of six ontologies for two other area taxonomy-related techniques. One is that concepts with larger number of relationship types have higher error rates than concepts with fewer number of relationship types [50]. Another is that if the top area has a large number of concepts, then a relatively large number of concepts are missing relationships [34]. An explanation of these two techniques is well beyond the scope of this paper.

## Conclusions

There is a need to achieve scalability in quality assurance of biomedical ontologies. We showed in this paper that for the large family of BioPortal ontologies with outgoing lateral relationships, concepts in small partial-areas of a partial-area taxonomy of an ontology have statistically significantly more errors than concepts of large partial-areas, for at least half of the ontologies in this family. To achieve this, we have shown this property for two hierarchies, the *Specimen* hierarchy of SNOMED CT and the *Gene* hierarchy of the NCIt in this paper. These two were added to the four other ontologies for which this property was established in previous research. Together they demonstrate the property for six out of six of the ontologies of this family.

## Data Availability

The datasets used and/or analysed during the current study are available from the corresponding author on reasonable request.

## Abbreviations

QA: Quality assurance
NCBO: National Center for Biomedical Ontologies
NCIt: National Cancer Institute Thesaurus
SNOMED CT: SNOMED Clinical Terms
GO: Gene Ontology
ChEBI: Chemical Entities of Biological Interest
SABOC: Structural Analysis of Biomedical Ontologies Center
OP: Object property
DP: Data property
DAG: Directed acyclic graph
OAF: Ontology Abstraction Framework

## Footnotes

1 The fact that both ontologies have 19 hierarchies is coincidental.
